# An accessible GenePattern notebook for the copy number variation analysis of Illumina Infinium DNA methylation arrays

**DOI:** 10.12688/f1000research.16338.1

**Published:** 2018-12-05

**Authors:** Clarence K. Mah, Jill P. Mesirov, Lukas Chavez

**Affiliations:** 1 Department of Medicine, University of California, San Diego, La Jolla, CA, 92093, USA; 2 Moores Cancer Center, University of California, San Diego, La Jolla, CA, 92093, USA

**Keywords:** Illumina Infinium methylation arrays, DNA methylation, copy number variation, pre-processing, interactive, visualization, GenePattern Notebook, Jupyter Notebook, open-source, conumee, minfi, R/Bioconductor

## Abstract

Illumina Infinium DNA methylation arrays are a cost-effective technology to measure DNA methylation at CpG sites genome-wide and across cohorts of normal and cancer samples. While copy number alterations are commonly inferred from array-CGH, SNP arrays, or whole-genome DNA sequencing, Illumina Infinium DNA methylation arrays have been shown to detect copy number alterations at comparable sensitivity. Here we present an accessible, interactive GenePattern notebook for the analysis of copy number variation using Illumina Infinium DNA methylation arrays. The notebook provides a graphical user interface to a workflow using the R/Bioconductor packages *minfi* and *conumee*. The environment allows analysis to be performed without the installation of the R software environment, the packages and dependencies, and without the need to write or manipulate code.

## Introduction

Although Illumina Infinium DNA methylation arrays, including the 450k and EPIC (“850k”) BeadChips, have been designed for detecting genome-wide DNA methylation, the resulting data can also be used to analyze copy number profiles ( Feber *et al.,* 2014). This feature allows the simultaneous analysis of DNA methylation and copy number variation (CNV) and reduces the quantity of material needed to perform both analyses. We have implemented an Illumina Infinium DNA methylation array-based CNV analysis workflow as an accessible, interactive GenePattern notebook, which integrates background information, workflow instructions, a graphical user interface, source code, and the results in a single electronic notebook document ( Mah, 2018). Leveraging the popular GenePattern Notebook environment ( Reich *et al.,* 2017), the notebook enables the sharing of reproducible analyses and results.

**Figure 1.**
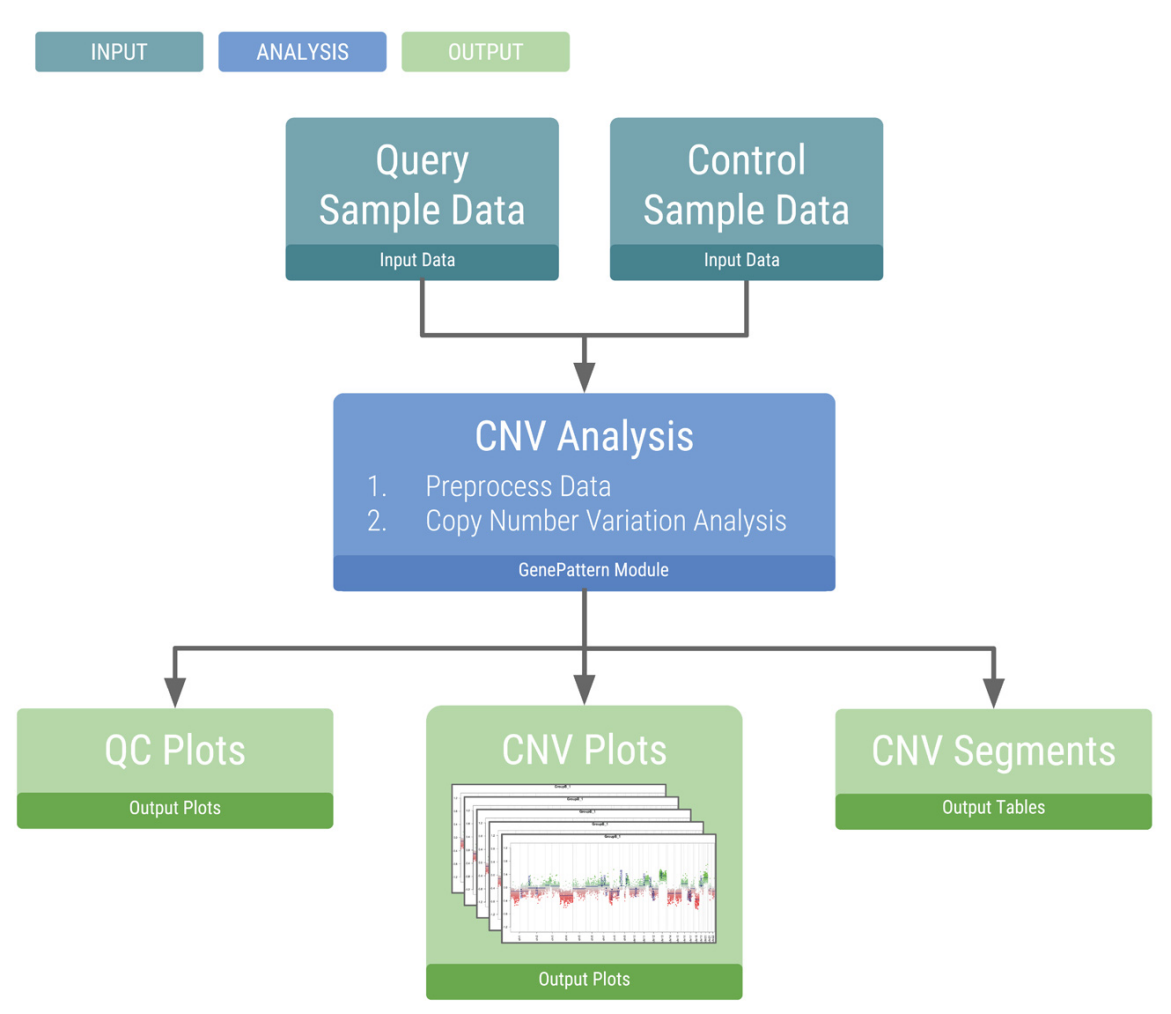
Analysis workflow. The flowchart shows the main inputs and outputs necessary for the copy number variation analysis.

The workflow is initiated by a single step and performs two main analyses: loading and preprocessing the data, and copy number analysis ( Figure 1). Multiple samples can be analyzed in parallel. The preprocessing step utilizes the *minfi* R package to load and process Illumina Infinium DNA methylation array data and to perform data normalization (Aryee, 2014). Copy number analysis is performed using the *conumee* R package, which compares each sample to a set of user-provided normal reference samples ( Hovestadt & Zapatka, 2015). This analysis outputs a set of copy number plots for the entire genome, individual chromosomes, and for user defined gene loci of interest. Copy number profiles are described as segments along the genome and can be exported as text files for visualization with tools such as the Integrated Genome Viewer ( Robinson *et al.*, 2011) and for further analysis.

## Methods

### Implementation

The entire workflow is implemented as a GenePattern notebook, which can be accessed at the GenePattern Notebook Repository ( http://www.genepattern-notebook.org/) and run there by the user. Data preprocessing and CNV analysis steps are implemented as a GenePattern module ( Reich *et al.*, 2006) and utilized by the *MethylationCNVAnalysis* notebook.

### Load and preprocess data

**Figure 2.**
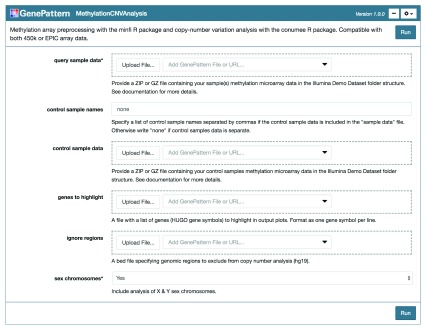
Copy number variation analysis GenePattern Notebook interface. The “MethylationCNVAnalysis” module is presented as an input form using the GenePattern Notebook graphical user interface. The user links or uploads input files and selects analysis parameters before pressing “Run” to execute the workflow.

To begin the analysis, two sets of data are required: the query sample data for which the copy number profiles are to be analyzed and appropriate control sample data used to establish baseline copy number profiles for comparison ( Figure 2). The input data for this notebook (query and control samples) are raw IDAT files generated by the microarray scanner, representing two different color channels prior to normalization. As described in the *minfi* documentation, IDAT files are the most complete data types, because they include measurements on control probes, which are necessary for assessing bisulfite conversion efficiency and for normalizing technical variability.

To load the Illumina Infinium methylation array data into the notebook, the IDAT files must be combined into a single archive (.zip or .gz formats). The archive can be organized either as a flat archive where all IDAT files are packed without subfolders, or as an archive in the standard folder structure as presented in the Illumina demo dataset. The IDAT archive can be selected and loaded through the graphical user interface of the GenePattern notebook. Both 450k array and EPIC array types are compatible as long as all samples in a single archive are of the same array type. If the query samples or control samples are of different array types, only the common set of probes between 450k and EPIC array types are evaluated across all samples.

**Figure 3.**
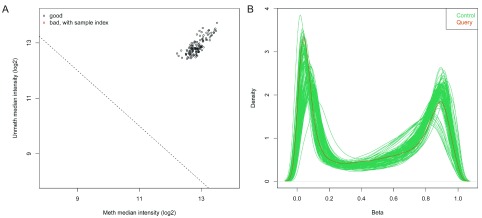
Plots of query and control samples. ( **A**) Median intensity plot of query & control samples. Log median intensity of the methylated channel is along the x-axis and log median intensity of unmethylated channel is along the y-axis. Bad-quality samples fall under the threshold and are colored red. There is no bad quality sample in this plot. ( **B**) DNA methylation (Beta-value) density plot of query & control samples. A density plot showing the distribution of beta values across each sample. Beta values should be bimodal and peak around 0 and 1.0.

For each sample, the data is normalized with respect to background and positive control probes on the arrays according to the implementation in Illumina’s proprietary GenomeStudio software. Upon loading the data, the notebook generates a quality control report containing two plots for identifying poor quality samples. The first plot shows the log _2_ median intensity of the methylated versus unmethylated channels ( Figure 3A). Poor-quality samples tend to have lower median intensities and separate from the good quality samples. The second plot shows the DNA methylation levels (Beta values) of all probes on the array and for all samples as a density plot in which we expect to see a bimodal distribution with peaks at zero (no methylation) and one (100% methylation) ( Figure 3B).

Control samples should be free of CNVs and have a similar methylation profile as the samples of interest. The best practice is to use control samples of the corresponding normal tissue type. If control samples are included in the query sample dataset, no separate data needs to be loaded. Instead, the control samples can be specified by providing the sample names in the CNV analysis step. Otherwise, the control data will be loaded as a separate archive of IDAT files.

### CNV analysis

As outlined in the *conumee* documentation, the copy number analysis is performed as follows: each query sample is normalized to the control samples by multiple linear regression yielding the linear combination of control samples that most closely fits the intensities of the query sample. Next, the log _2_ ratio of probe intensities of the query sample versus the combination of control samples are calculated. Probes are then combined within predefined genomic bins. Intensity values are shifted to minimize the median absolute deviation of all bins to zero to determine the copy-number neutral state. The genome is segmented into regions of the same copy number state using the circular binary segmentation algorithm ( Seshan & Olshen, 2018).

Genomic loci of genes to be highlighted in the CNV plots are retrieved from the hg19 Ensembl database using the BiomaRt R package (Durinck, 2005; Durinck, 2009). The notebook also offers an option to exclude regions from analysis, such as highly polymorphic regions that would yield inaccurate copy number calls. In addition, X and Y chromosomes can be excluded to avoid misleading results in case no appropriate control data is available.

### Operation

To run the *MethylationCNVAnalysis* notebook, the user must have a GenePattern account that can be created on the GenePattern Notebook website ( http://genepattern-notebook.org). After logging in, the notebook can be found in the “Community” section of the “Public Notebooks” page. The notebook can then be run from the GenePattern Notebook site, with no additional software installations needed.

## Use case

The use case presented by the notebook evaluates the copy number profile of a glioblastoma tumor analyzed by an Illumina Infinium 450k DNA methylation array. This sample has been classified as an IDH wild-type midline glioblastoma according to the methylation-based classifier described by Capper *et al.* (2018). Recurrent chromosomal alterations of this tumor type are gain of chromosome 7 with or without EGFR amplification (>80%), loss of 9p21 (CDKN2A/B; >50%) and chromosome 10 loss (>70%). Amplifications of the PDGFRA oncogene are enriched in this class (present in 20–30% of cases) (Capper, 2018).

**Figure 4.**
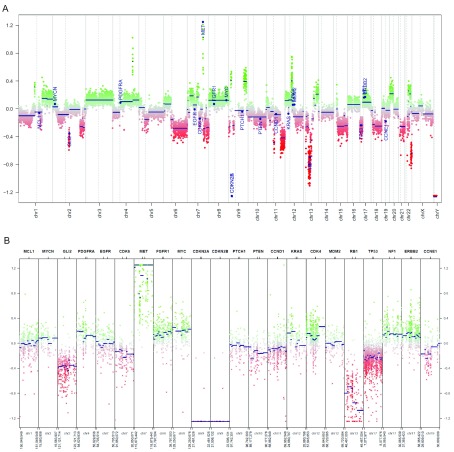
Plots of copy numbers. ( **A**) Copy number plot of the entire genome in the example glioblastoma sample. A plot of all chromosomes across the genome. Intensity values of each bin are plotted as colored dots, green indicating above normal copy number, red indicating below normal copy number, and grey indicating close to normal copy number. Blue lines indicate the median intensity of each bin. Specified genes to be highlighted are annotated. ( **B**) Copy number plot of common cancer genes in the example glioblastoma sample. An overview of the genomic loci of common cancer genes are shown in more detail. Copy number values are visualized as described in Figure 4A.

We used the 450k methylation profiles of 119 normal brain tissue samples as the corresponding control data (Capper, 2018). By inspecting the generated CNV plots, we can visually identify significant copy number loss of CDKN2A/B relative to normal brain tissues ( Figure 4). Additionally, several copy number changes that are associated with glioblastoma stand out, notably MET amplification and loss of RB1.

## Conclusion

The GenePattern notebook *MethylationCNVAnalysis*, hosted in the GenePattern Notebook Repository, processes Illumina Infinium DNA methylation array data and generates CNV segments and plots. Different designs of Illumina Infinium DNA methylation arrays have been produced by the manufacturer including the 450k and EPIC arrays. Importantly, different batches of these designs can contain a variable set of probes. As a result, the GenePattern notebook requires all query samples to be of the same array design. Similarly, all control samples have to be of the same array design, which can be different from the query samples. If the query samples and the control samples are of different array designs, only the common set of probes between the array designs are evaluated for the CNV analysis. As described above, the choice of control samples is crucial for the resulting copy number profiles. The control samples should be free of CNVs and have a similar methylation profile as the samples of interest. Provided that query and corresponding control samples are available, the *MethylationCNVAnalysis* notebook in the GenePattern Notebook Repository allows the CNV analysis to be performed without the installation of software and without the need to write or manipulate code.

## Data availability

The notebook includes links to the data for running the use case described above. The raw data can be found in GEO Series GSE90496: https://identifiers.org/geo/GSE90496.

## Software availability

GenePattern Notebook is available from: http://genepattern-notebook.org/.

A public preview of the notebook is available from: https://notebook.genepattern.org/services/sharing/notebooks/136/preview/

GenePattern Notebook source code is available from: https://github.com/genepattern/methylation_cnv_analysis_notebook.

Archived source code at time of publication: https://doi.org/10.5281/zenodo.1419319 ( Mah, 2018).

License: BSD 3-Clause.
