# Supplementary material for: An accessible GenePattern notebook for the copy number variation analysis of Illumina Infinium DNA methylation arrays
Source: F1000Res. 2018 Dec 5;7:ISCB Comm J-1897. [Version 1] doi: 10.12688/f1000research.16338.1 (PMC6498745; doi:10.12688/f1000research.16338.1)
Supplement: Peer review history

# Peer review: Reviewer response for version 1

**DOI:** 10.5256/f1000research.17848.r47252

**Published:** 2019-05-02

**Authors:** Aris Floratos

**Affiliations:** 1 Department of Systems Biology, Columbia University, New York, NY, USA

The article describes a GenePattern notebook for inferring CNVs using methylation profiling data generated by Illumina Infinium arrays. The notebook leverages the well-established Bioconductor packages minfi and conumee to implement an analysis workflow that comprises quality control, CNV calling, and results visualization. Functionality is made available through a web browser interface and requires no software installation/configuration, making it an attractive option for users with limited informatics expertise.

 Some thoughts about possible improvements:

1. Given that the entire workflow can be somewhat time consuming (the notebook documentation indicates that processing a single sample takes about 2 minutes), it would be useful if there was an option to run the QC step as a stand-alone computation, not combined with the CNV calling. As things stand right now, a significant amount of time can be spent waiting for the analysis to complete, only to realize that some control samples are of low quality and, thus, need to be removed and the analysis be rerun.
2. When running the notebook without specifying values in the “genes to highlight” or “ignore regions” parameter boxes, the run fails with “getopt” error messages. It is not clear why these parameters are mandatory (e.g., in the case of "genes to highlight", it is conceivable that one may want to only inspect genome-wide patterns of aberration, without focusing on specific genes); but if so, it would be helpful to state clearly in the documentation section.

I have read this submission. I believe that I have an appropriate level of expertise to confirm that it is of an acceptable scientific standard.

# Peer review: Reviewer response for version 1

**DOI:** 10.5256/f1000research.17848.r45332

**Published:** 2019-04-15

**Authors:** Robert Ivanek

**Affiliations:** 1 Department of Biomedicine, University of Basel, Basel, Switzerland

The article by CK Mah et al. describes a new GenePattern Notebook "MethylationCNVAnalysis" which allows users of GenePattern platform to run copy number analysis (CNV) of Illumina Infinium DNA methylation arrays. The tool provides graphical user interface to the CNV analysis based on Bioconductor packages `minfi` and `conumee`. Such analysis can be with this tool performed even without programming experience or without functional R installation.

 Compared to analysis on the R command line, the user is asked to set only few parameters required for the analysis: set of test and control samples, gene list for detailed view, a black-list of regions excluded from the analysis and a coice to in-/exclude sex chromosomes. In my opinion it would be helpful to create a section with "Advanced settings" and allow also specification of few other parameters: `bin_minprobes` and `bin_minsize` for function `CNV.create_anno` from `conumee` package and parameters for segmentation of log2ratio (functions from `DNAcopy` package). In both cases the authors of `conumee` package warn, that they optimized parameters for 450k arrays and another array type might require further optimization (see http://bioconductor.org/packages/release/bioc/vignettes/conumee/inst/doc/conumee.html). Also in respect to general need for higher reproducibility of analyses, the tool should export short summary of software, their versions and parameters used for analysis.

I have read this submission. I believe that I have an appropriate level of expertise to confirm that it is of an acceptable scientific standard.
